# Adsorption of Wine Constituents on Functionalized Surfaces

**DOI:** 10.3390/molecules21101394

**Published:** 2016-10-18

**Authors:** Agnieszka Mierczynska-Vasilev, Paul A. Smith

**Affiliations:** The Australian Wine Research Institute, P.O. Box 197, Glen Osmond, Adelaide 5064, South Australia, Australia; paul.smith@awri.com.au

**Keywords:** wine, adsorption, functionalized surfaces, plasma polymers

## Abstract

The adsorption of macromolecules on solid surfaces is of great importance in the field of nanotechnology, biomaterials, biotechnological, and food processes. In the field of oenology adsorption of wine macromolecules such as polyphenols, polysaccharides, and proteins is much less desirable on membrane materials because of fouling and reduced filtering performance. On the other hand, adsorption of these molecules on processing aids is very beneficial for achieving wine clarity and stability. In this article, the effect of surface chemical functionalities on the adsorption of white, rosé, and red wine constituents was evaluated. Allylamine, acrylic acid, and ethanol were selected as precursors for plasma polymerization in order to generate coatings rich in amine, carboxyl, and hydroxyl chemical groups, respectively. The surface chemical functionalities were characterized by X-ray photoelectron spectroscopy (XPS) and the ability of different surface chemical functionalities to adsorb wine constituents were characterized by quartz crystal microbalance with dissipation (QCM-D) and atomic force microscopy (AFM). The results demonstrated that the amine and carboxyl modified surfaces encourage adsorption of constituents from white wine. The hydroxyl modified surfaces have the ability to preferentially adsorb rosé wine constituents, whereas red wine adsorbed to the highest extent on acrylic acid surface.

## 1. Introduction

Adsorption of macromolecules on solid surfaces and the changes in surface properties caused by macromolecules adsorption are major concerns in a number of fields, such as biology, medicine, biotechnology, and food processing [1,2,3]. For example, protein solutions, liquid foods containing proteins or microbial suspensions are on numerous occasions treated by ultrafiltration and reverse-osmosis for concentration or dialysis purposes. The main problem in these techniques is the adhesion of proteins onto the membrane surface [4]. Similarly, in food and bio-processes including those that use bioreactors, proteinaceous soils adhere to the wall surface of the equipment or pipes. The adherence behavior of soil particles on the solid surfaces affects the efficiency of detergents during the cleaning process [5]. A strong solid-liquid interfacial activity is exhibited by plant polyphenols [6,7]. For example, catechols (high dihydroxyphenyl) and gallic acid (trihydroxyphenol)—constituents of marine polyphenolic protein adhesives—are known to bind strongly to surfaces through covalent and noncovalent interactions [8]. Tannic acid is, on the other hand, polyphenolic constituent responsible for tea staining and tanning with substrate-independent adsorption ability [9]. Various surface modification techniques were developed in the past 30 years such as chemical and ozone-induced grafting, physical adsorption, plasma deposition, radiation, Langmuir-Blodgett deposition, or self-assembly technology [10,11,12]. The modifications of surface wettability, hydrophobicity and surface charge were shown to alter the extent of protein adsorption, for example [13]. Recently, more effort has been placed on creating uniformly coated surfaces with different chemical functionalities in an attempt to determine more specifically what surface chemical properties, beyond hydrophobic or hydrophilic characteristics, can improve biomaterial compatibility [14,15,16,17]. 

In wine industry, many significant and unresolved problems such as selective removal of undesired constituents from wines—i.e., removal of smoke taint compounds, bitter molecules, haze forming proteins, or managing deposits from the surface of the manufacturing equipment for maintains or sanitary conditions [18]—can be successfully resolved if wine constituents adsorption is predicted and controlled. Such knowledge has a potential to substantially reduce losses and improve performance of wine operations.

Our previous work described the role of functional groups (amine, ammonium, carboxyl, sulfonate, methyl, hydroxyl, and formyl) in adsorption of red wine constituents before and after filtration [19]. In the present study, we use the same approach to investigate the effects of three functional plasma-polymerized coatings (amine, carboxyl, and hydroxyl) on white, rosé, and red wine adsorption.

It is well known that different grape varieties and yeast strains make different types of wines. Since wines vary in their chemical composition our hypothesis is that different wine components from different wines will adhere on the various functionalized surfaces. For this purpose, allylamine, acrylic acid, and ethanol were selected as precursors for plasma polymerization in order to generate coatings rich in amine (–NH_2_), carboxyl (–COOH), and hydroxyl (–OH) chemical groups, respectively.

Plasma polymer deposition is a very attractive surface modification technique [20] which can effectively generate an ultrathin and pinhole free film with high retention of functional groups and uniform surface coverage [21]. Unfractioned wines were directly monitored during adsorption on plasma polymer surfaces and subsequently characterized by XPS and AFM. 

In this study, a combination of QCM-D and AFM was used to characterize the adsorption of white, rosé, and red wine on substrates of different surface chemistries. The combination of those two techniques was used for determination of adsorbed wine mass and the viscoelastic properties of wine layers. A knowledge generated about the adsorption behavior of wine constituents at functional plasma-polymerized surfaces is valuable for better understanding of intermolecular interactions at surfaces.

## 2. Results

### 2.1. NTA Measurements of Particle Size and Concentration

**Figure 1:**
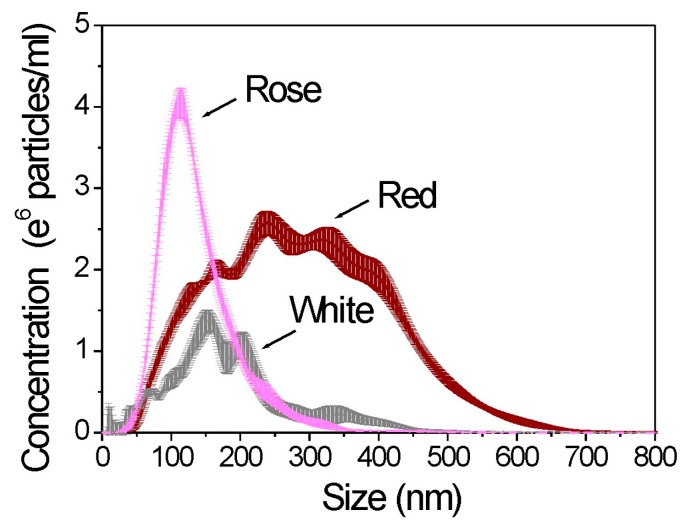
Particle size distributions of white, rosé, and red wine samples.

Nanoparticle tracking analysis (NTA) was used for the assessment of particle size and concentration [22]. Figure 1 shows that NTA does not have problems with polydispersity. It can resolve and accurately measure different size particles simultaneously within the same solution. NTA also allows the particle concentration to be estimated directly. The NTA results (total concentration, standard deviation, mean size, and d90) for all studied wine samples are shown in Table S1 (see Supplementary Materials). The greatest total particle concentration was 8.41 E8/mL at the highest particle size of 292 nm for the red wine sample. The percentile undersize of diameter at 90 nm (d90) for red wine was 454 nm. Interestingly, red wine had also the most polydisperse size distribution profile with the size distribution range between 45 to 700 nm as compared to white and rosé wines. By way of comparison the second greatest total particle concentration of 4.05 E8/mL was at 140 nm for rosé wine. The rosé wine sample was characterised by the d90 of 211 nm.

White wine sample had the lowest particle concentration (2.04 E8/mL), the d90 was 327 nm and the mean diameter was 190 nm. The data suggest that there is a significant difference in particle characteristics such as d90, total particle concentration, and mean particle diameter between all studied wine samples. Overall, the results of the study demonstrated that there was a decrease in d90 and mean particle diameter in the following order: red wine > white wine > rosé wine. On the other hand, there was a decrease in total particle concentration in the following order: red wine > rosé wine > white wine.

### 2.2. Surface Chemistry

#### 2.2.1. Acrylic Acid Plasma Polymer (ppAcrA)

**Figure 2:**
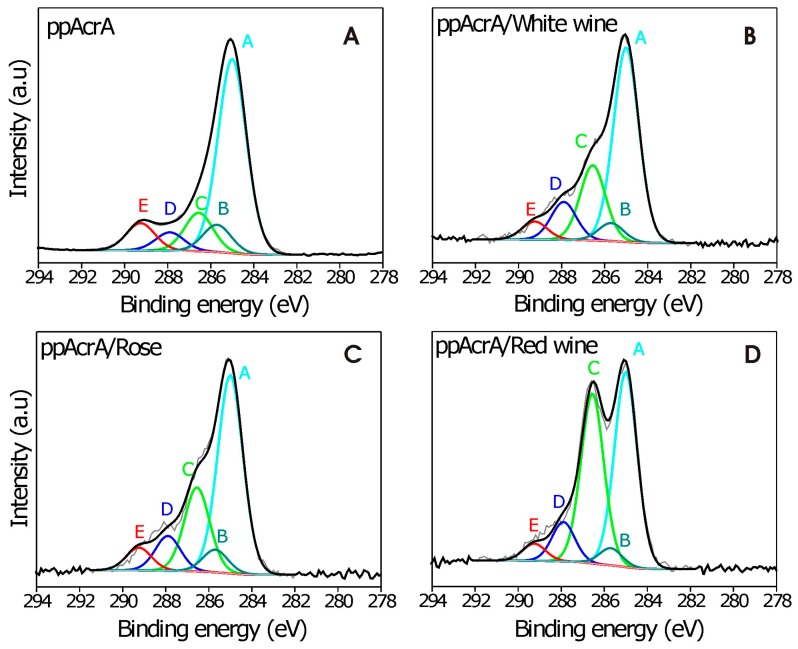
High resolution C1s spectra of different samples: (**A**) acrylic acid plasma polymerized surface (ppAcrA); (**B**) white wine; (**C**) rosé wine; and (**D**) red wine after adsorption on ppAcrA surface.

Plasma polymerized films from acrylic acid, allylamine, and ethanol were produced and analyzed using XPS. The survey spectra were used to obtain the surface O/C and N/C content of the plasma polymers and the C1s core levels were fitted to obtain information about functional groups. The XPS survey spectra of ppAcrA revealed only carbon (76.17%) and oxygen (23.83%) in the deposits (Figure S1 in Supplementary Materials). The C1s core level spectra of the ppAcrA coatings were peak fitted for various oxygen-containing functionalities as shown in Figure 2A. First, spectra were corrected for sample charging, setting the hydrocarbon signal to 285 eV (peak A). The following functionalities were then fitted: alcohol/ether (C–OH/R) = peak B at a shift of +1.55 eV (286.55 eV), carbonyl (C=O) = peak C at +2.9 eV (287.9 eV), carboxylic acid/ester (COOH/R) = peak D at +4.25 eV (289.25 eV), and a β-shifted carbon bonded to carboxylate (C–COOH/R) = peak E at +0.7 eV (285.7 eV) [23,24,25].

The effect of the carboxyl functional group on wine adsorption was also investigated. Three wines were brought into contact with the QCM sensors coated with acrylic acid plasma polymer. After 40 min of adsorption, washing with MilliQ water was performed to remove any non-surface-bound wine material and later on, the XPS was performed on these samples. The high resolution C1s spectra for wine-bound surfaces are shown in Figure 2B–D. A five-component fitting routine was employed. For ppAcrA/wine coated samples the C1s spectra show higher proportion of the carbonyl/amide (peak C) and the alcohol/ether/imine components (peak D). This can be attributed to the imine and amide bonds present in surface-bound wines as well as an increase in alcohol, ether and carbonyl bonds. Interestingly, the C component has much higher proportion for red wine, followed by rosé and white wine, whereas the D component has a higher proportion for white wine, followed by rosé and red wine. Further evidence for wine molecule attachment was found by collecting the high resolution N1s spectra (Figure S2 in Supplementary Materials). The XPS spectra of ppAcrA had no traces of nitrogen in the N1s spectra of the acrylic acid, which indicated that no N contamination was present, but the ppAcrA coated with different wines exhibited nitrogen peaks.

**Figure 3:**
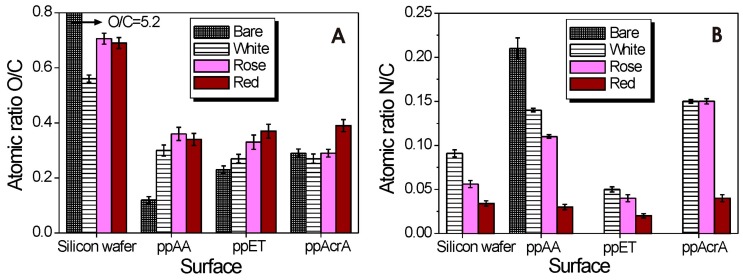
Surface (**A**) O/C and (**B**) N/C content of wines simply dried on a silicon wafer and after adsorption onto plasma polymerized surfaces.

The O/C and N/C atomic ratios before and after the adsorption of wines are presented in Figure 3. In addition, for the purpose of comparison, the O/C and N/C ratio was also calculated for the wine samples simply dried on the silicon wafer. The above comparison between surfaces help us realize that the O/C ratio almost doubled in the case of the wine samples directly dried on the silicon wafer as compared to the wine adsorbed on functionalized surfaces, no matter the functionality. It is worth mentioning that the O/C ratio of bare silicon wafer is high (5.2 ± 0.7) when compared to all other surfaces and decreases after wine adsorption.

On the other hand, the N/C ratio substantially increased on ppAcrA and ppAA surface after white and rosé wine was adsorbed and almost did not change its value after red wine adsorption on ppAcrA and ppAA surfaces when comparing with wine samples directly dried on the silicon wafer. In the case of ppET surface, the N/C ratio decreased regardless of the wine.

When the ratios are compared with bare plasma polymerized surfaces it can be seen that the O/C ratio almost did not change after white and rosé wine adsorption and increased on the ppAcrA red wine coated sample as compared to bare ppAcrA coating (Figure 3A). The N/C ratio increased after wine adsorption, with rosé and white wines having the same high ratio (Figure 3B). The presence of nitrogen in the XPS spectrum is evidence of the nitrogen compounds present in wines.

#### 2.2.2. Allylamine Acid Plasma Polymer (ppAA)

The XPS survey scans were also made of plasma-polymerized surfaces prepared from allylamine. Those deposits contained carbon (75.07%), oxygen (8.95%), and nitrogen (15.99%). The XPS data were quantified and the results are shown in Figure S3A (Supplementary Materials). The C1s core level spectra of the ppAA were peak fitted for nitrogen-containing functionalities, using chemical shift values reported in the literature [23,24]. The functionalities fitted were: amine (C–NR2) at +0.9 eV (285.9 eV), imine (C=N) at +1.7 eV (286.7), and amide (CNO) at +3.0 eV (288.0 eV). 

To verify wine molecules and wine colloids binding to ppAA surfaces the high resolution C1s spectra were obtained by XPS. The ppAA/wine C1s peak can be fitted with six components as shown in Figure S3B–D (Supplementary Materials). Figure 3 presents the ratio of O to C and N to C in allylamine pp films after wine adsorption. The results indicate the decrease in O/C ratio for wine coated samples in the following order: red > rosé > white wine. The N/C ratio showed a decrease for the wine treated surfaces as compared to bare ppAA (Figure 3).

#### 2.2.3. Ethanol Acid Plasma Polymer (ppET)

The XPS survey spectra of ppET revealed only carbon (81.63%) and oxygen (18.37%) in the deposits. The O/C ratio was measured and is shown in Figure 3. The C1s core level spectra of the ppET coatings were peak fitted for various oxygen-containing functionalities. Like in the previous two cases spectra were corrected for sample charging. As demonstrated in Figure S4 (Supplementary Materials), the C1s peak of ppET/wine can accommodate, as observed for bare ppET, five components. In the bare ppET, the O/C ratio determined from the C1s and O1s peak areas is 0.23. The O/C ratios from ppET/wine samples are given in Figure 3. Higher ratio was observed for red wine, intermediate for rosé and the lowest ratio for white wine. There was an increase in the N/C ratio for surfaces after wine adsorption as compared to bare ppET.

### 2.3. Contact Angle Measurements

**Figure 4:**
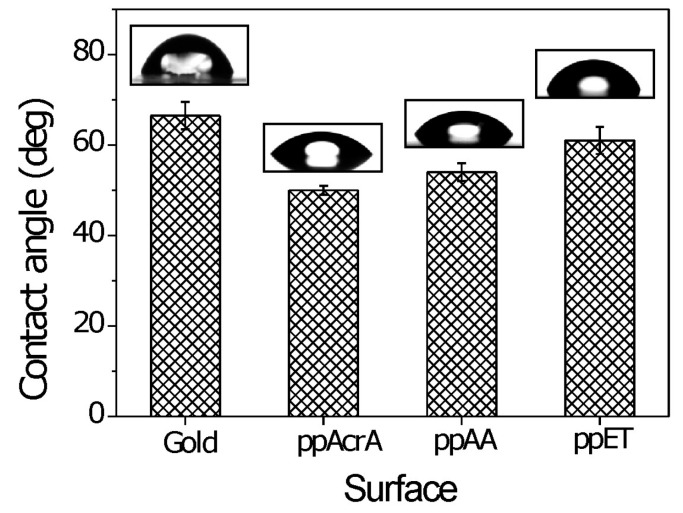
Static water contact angle of uncoated gold surface and pp coatings. Representative images of water droplets on three different samples are placed above the contact angle values. Measurements were made on five drops and averaged.

The static water contact angles on the surfaces of uncoated gold sensor and pp coatings are shown in Figure 4. PP coatings of the sensors led to decreased surface wettability. After coating the contact angles changed from 66.5° for the uncoated gold sensors to 50°, 54°, and 61° for ppAcrA, ppAA, and ppET, respectively. Overall, the measured contact angles are in line with recent literature values [25,26]. The results provide an evidence that the changes in surface chemical composition exert influence on wettability of the material and hence strongly correlates with the interfacial interactions [27].

### 2.4. Quantification of Bound Wine

The gold-coated quartz crystals were first treated with acrylic acid, allylamine, and ethanol plasma in a glow-discharge apparatus in order to deposit carboxyl, hydroxyl, and amino groups on their surfaces. Then the coated crystals were exposed to three different wines (white, rosé, and red) in QCM-D experiments. Due to the large change to the dissipation energy loss observed during measurements (Figure S5 in Supplementary Materials), Voigt-based viscoelastic modeling was required to accurately determine the adsorbed mass values [28]. The agreement between the experimental data and the model was very good as presented in Figure S6 (Supplementary Materials).

**Figure 5:**
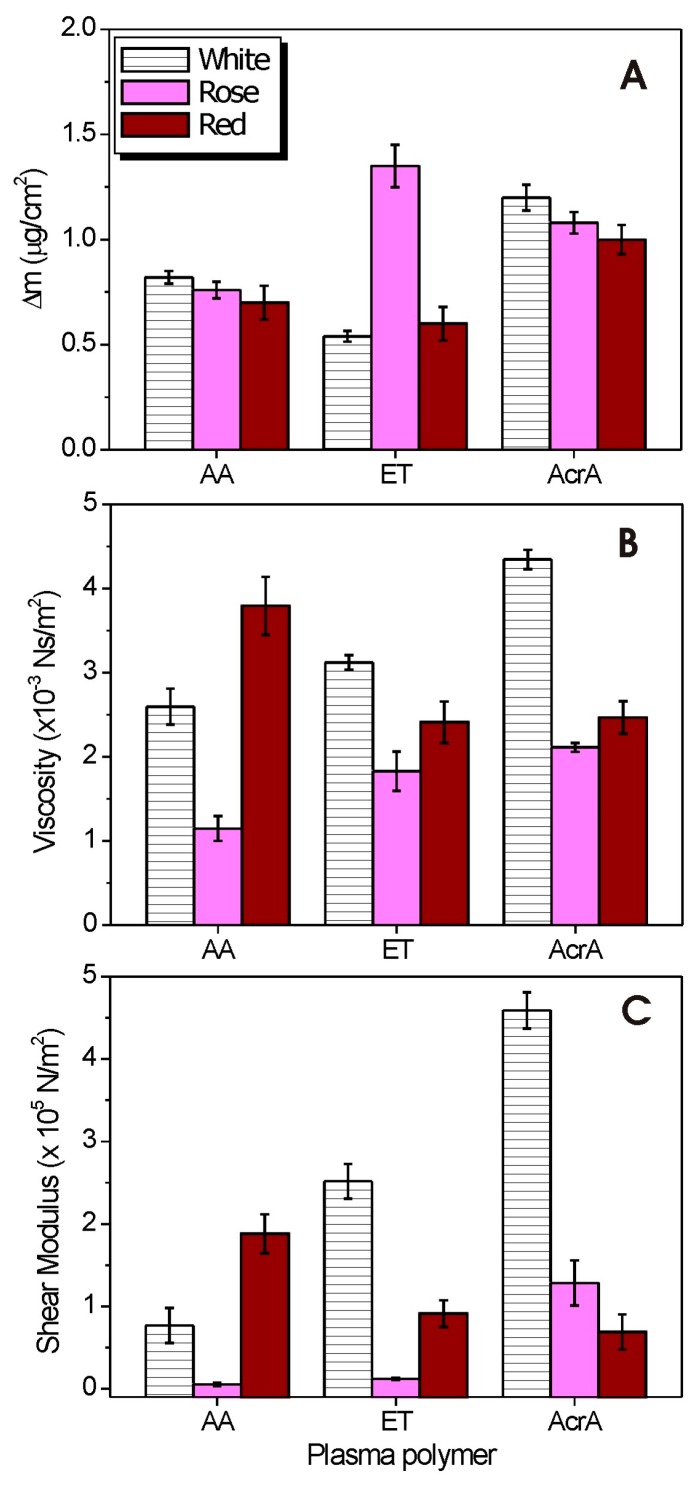
(**A**) Mass changes; (**B**) viscosity changes; and (**C**) shear elastic modulus of various wine samples on the pp surfaces estimated from the Voigt model.

Figure 5A shows the comparison of the mass changes (Δm) for the three different wines in three different environments.

The levels of mass uptake of wine molecules on three different surfaces are distinct. On –NH_2_ modified substrates the adsorbed amount of white and rosé wine are higher than red wine. The trend was similar on –COOH modified substrates (white > rosé > red wine) but adsorbed mass was much higher than on –NH_2_ modified substrates. Finally, on –OH modified substances the wine mass uptake was as follows: the highest for rosé wine, followed by red wine and white wine. 

Figure 5B,C show the variations of viscosity and shear elastic modulus of the three different samples calculated from the Voigt model adsorbed onto three different pp surfaces. On the ppAA surface both the viscosity and the shear elastic modulus values of the investigated red wine sample is larger as compared to white and rosé wines. On the ET surface, the white wine is much more viscoelastic then the rosé and red wine. On the AcrA surface, white wine is more viscoelastic as compared to other wines, whereas the red wine is quite viscous but with rigid conformation. Possible reasons for the differences in the adherence and viscoelasticity of the adsorbed wine on the three investigated surfaces are differences in the mass concentration on the pp surfaces, different levels of wine molecules and wine colloids at different locations in the pp coatings, as well as variations in strengths of intermolecular forces between the molecules on the surface.

### 2.5. Atomic Force Microscopy

#### 2.5.1. Topography

Topography of bare pp surfaces as well as adsorbed wine constituents on pp surfaces was assessed through tapping mode Atomic Force Microscopy (TMAFM, NT-MDT SPM, Moscow, Russia). The AFM height and phase images of the bare QCM sensor (Q-Sense, Västra Frölunda, Sweden) are depicted in Figure S7. The QCM gold sensor’s surface is characterized by an rms roughness value of 3.33 nm as summarized in Table S2 (Supplementary Materials). The AFM topographic images show that the surface of the gold surface is composed of well-oriented and interconnected structures containing irregular short channels or grooves (typical for a gold surface).

**Figure 6:**
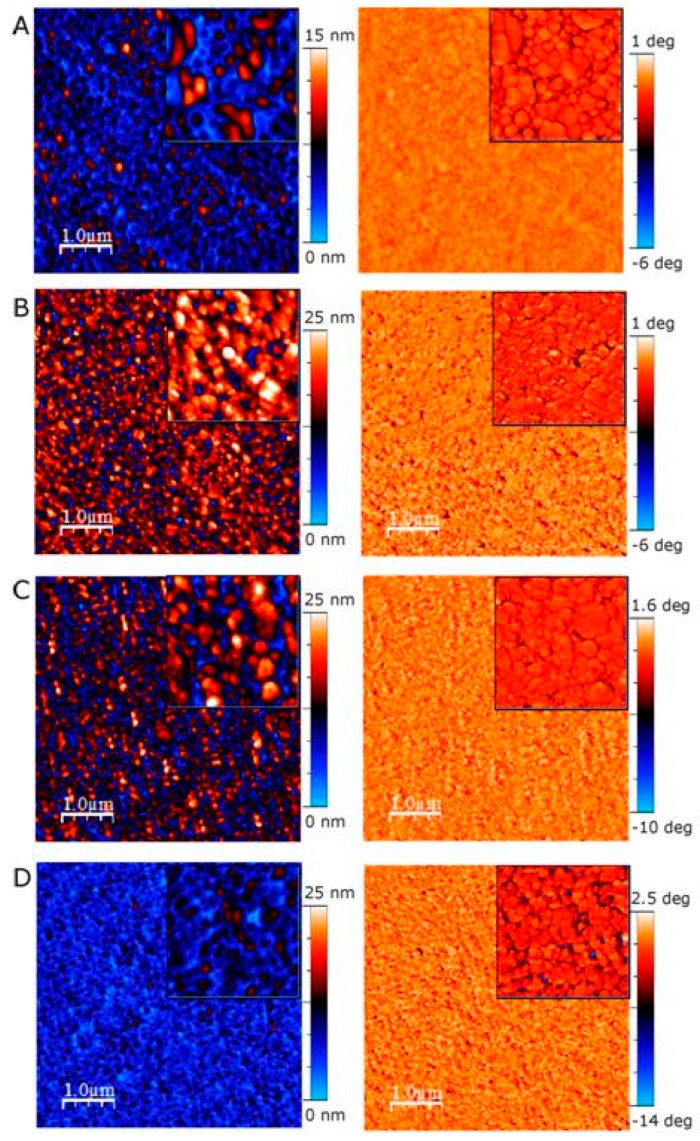
5 μm × 5 μm TMAFM images (left: height images; right: phase images) of: (**A**) bare AcrA pp surface; (**B**) white wine; (**C**) rosé wine; and (**D**) red wine on ppAcrA. Inserts are 2 μm × 2 μm AFM images.

Figure 6A shows the ex situ AFM height and phase images of pp-acrylic acid deposited on gold QCM sensor (Q-Sense). The ppAcrA is composed of well-oriented and interconnected structures replicating the morphology of the gold substrate. The RMS roughness decreased from 3.33 to 1.65 nm. Figure 6B–D shows the representative height and phase TMAFM images of white, rosé, red wines adsorbed on acrylic acid surface. In all three cases the morphology is similar, replicating the morphology of the substrate. The difference between the samples is the rms roughness and the layer thickness. As presented in Table S2 (Supplementary Materials) white wine adsorbed on ppAcrA surface with the highest values of rms roughness and peak-to-valley distance, followed by rosé and red wine. Those results are in agreement with the results from the QCM experiments which showed higher mass uptake for white wine as compared to rosé and red wine. Ex situ height and phase images of wine constituent adsorption was also taken on two other pp surfaces. The AFM images are shown in Figures S8 and S9 for allylamine and ethanol plasma polymer surface, respectively (see Supplementary Materials for details).

#### 2.5.2. Water Content

The combination of the QCM-D and AFM allowed to get an estimation of water content in the adsorbed wine layers. The volume of adsorbed wine over the AFM image area can be estimated from the AFM images by multiplying the average layer thickness by the area fraction of the wine coverage [21]. Calculated this way, volume per unit area can then be compared with the volume of wine adsorbed from the QCM-D experiments. As shown in Table 1, the adsorbed amount of wine per unit area in the AFM images is lower than that determined from the QCM-D. The most likely cause for the differences in adsorbed wine volume is that the features observed in the AFM images excludes hydration water attached to the wine constituents, making the imaged volume much smaller than the volume of actual adsorbed wine material (images are taken ex situ). The observation of hydration water in the adsorption of macromolecules is not unusual [29].

As shown in Table 1, the amount of hydration water for all investigated wine samples is found to be between 55% to 89% of the total adsorbed mass. The high values of hydration water are similar to previous QCM-D determination of the adsorbed layer water content for polysaccharides [30,31]. Also, proteins form highly hydrated layers (94% water) [32]. The highest amount of hydration water is observed for ppET/white wine (89%) whereas, the least water content within the adsorbed wine layer formed is found for ppAcrA/rosé (55%). Most likely the plasma polymer surface influences amount of hydration water of the wine layers, possibly through alternation of the adsorbed wine layer conformation when adsorbed on the different plasma polymer surfaces.

## 3. Discussion

### 3.1. Adsorption on ppAA

The amine group is protonated at low pH values thus leading to a more hydrophilic surface. As pH increases, deprotonation makes the surface neutral and its wettability decreases. As we measured previously by colloid probe AFM, due to the complete protonation of the NH_2_ groups at pH 3, the surface potential at the ppAA surface is high and positive (210 mV) [33]. The XPS results indicated the increase in O/C ratio for the wine constituents bound surfaces whereas, the N/C ratio showed a decrease for the wine treated surfaces as compared to bare ppAA. The O/C ratio is a result of carbon containing constituents’ adsorption onto the amine plasma polymerized surface while the N/C ratio is a result of nitrogen containing constituents to be present on the ppAA surface. The positively charged amine surface attracts negatively charged carbon-containing wine constituents—e.g., acidic polysaccharides [34] and condensed tannins—as demonstrated by the increase of O/C ratio from 0.12 to 0.30, 0.34, and 0.36 for white, rosé, red wine, respectively (please refer to Figure 3A in the manuscript). The increased values of the O/C ratios correlate very well with measured wine total polysaccharides and total tannin concentrations. For example, the highest polysaccharide content has red wine (520 mg/L), followed by rosé (315 mg/L) and white wine (222 mg/L). While red wine has the highest amount of wine tannins (909 mg/L), rosé has a little amount of tannin (57 mg/L) and in white wine the tannins were not detected (as given in Table S3 in Supplementary Materials). The negatively charged wine constituents are likely to adsorb onto positively charged surface due to the electrostatic interactions. It is also possible that some proteins are immobilized on the amine-terminated surface through the formation of an interfacial amide bond between the surface amine groups and carboxyl groups on the protein. On the –NH_2_ modified surfaces the highest adsorbed amount was obtained for white wine (12 mg/m^2^) followed by rosé (9 mg/m^2^) and red wine (7 mg/m^2^). According to the wine analysis, white wine has the highest protein concentration (134 mg/L), does not contain tannin and has the lowest polysaccharide content (222 mg/L). Rosé wine has a little protein (only 5 mg/L), some tannin (57 mg/L), and more polysaccharides than white wine (315 mg/L). Finally, red wine has the highest polysaccharide (520 mg/L) and tannin content (909 mg/L) and only little protein (red wines contain small amounts of free protein). 

The above results indicate that there are at least two possible mechanisms of adsorption onto amine surface: (1) electrostatic adsorption between positively charged amine surface and negatively charged wine constituents (acidic polysaccharides and condensed tannins); and (2) covalent immobilization of wine proteins onto amine surface.

### 3.2. Adsorption on ppAcrA

The zeta potential of carboxyl-rich surface is neutral at pH 3 and became negative with increasing pH, which is consistent with the weak acidity of –COOH groups [33]. At wines pH (Table S3 in Supplementary Materials) the –COOH groups are largely protonated and thus there is a little negative charge on the AcrA pp surface (0 charge at pH 3 and around −15 mV at pH 3.6) as measured previously by colloid probe AFM [33]. Whereas the XPS spectra of ppAcrA had no traces of nitrogen in the N1s spectra of the acrylic acid, the ppAcrA coated with different wines exhibited a nitrogen peak. This observation is the evidence that some of the nitrogen containing compounds—e.g., proteins adsorbed onto ppAcrA surface. Plasma-generated carboxylated surfaces are known to provide a convenient platform for the interfacial immobilization of molecules that contain amine groups, such as proteins [35]. Proteins, amino acids, and peptides make up the nitrogenous fraction of must and wines. The carboxyl modified surfaces promote white wine adsorption with adsorbed amount value of 15 mg/m^2^. As mentioned previously and presented in Table S3, among all investigated wines, white wine contains the highest amount of protein (137 mg/L), followed by rosé (5.25 mg/L) and red wine.

### 3.3. Adsorption on ppET

The hydroxyl groups are neutral and hydrophilic. They are less attractive than amines and carboxyls as platforms for covalent immobilization of biomolecules in aqueous solution due to their lower nucleophilic character and thus lower chemical reactivity. The –OH groups can bind, for instance, a range of peptides without any crosslinking or coupling agent which appears to be a physisorption rather than, for example, covalent interfacial linkage. The QCM-D demonstrates that the ppET enhanced rosé wine adsorption with the highest adsorbed amount of 17.5 mg/m^2^ among all investigated wine samples. The peak fit of rosé wine bound ppET surface revealed that 22.85% of the carbon was in alcohol/ether and imine environment, 8.75% of the carbon was in carbonyl and amide environment and 3.46% of the carbon was in a carboxylate-type environment (carboxylic acid or ester).

## 4. Materials and Methods

### 4.1. Wines

Three wines were selected for this study: 2012 AWRI sequential harvest trial Shiraz (Red), 2014 unfined SA Riesling (White) and 2014 Pinot Noir from Adelaide Hills (Rosé). 

Basic chemistry of the wines—such as alcohol, pH, titratable acidity, glucose/fructose, and volatile acidity—were analyzed by the AWRI Commercial Services using a Foss WineScan FT 120 and is provided in the Table S3 (see Supplementary Materials). The aspiration method was used to measure free and total SO_2_. The methyl cellulose precipitable (MCP) tannin assay was used to measure the total wine tannin in red and rosé wine sample, whereas phenol-sulphuric acid assay was used to measure total polysaccharides concentration in wines. Proteins in wines were analyzed by HPLC method [36] with some modifications. Separation was achieved using Agilent 1260 UHPLC with a Prozap Expedite C18 column, a solvent system of 0.1% TFA/H_2_O (Solvent A) and 0.1% TFA/ACN (Solvent B), at 0.75 mL/min and 35 °C column temperature. The mobile phase gradient was: 0–1 min 10%–20% Solvent B, 1–4 min 20%–40% B, 4–6 min 40%–80% B, 6–7 min 80% B, 7.01 min 10% B, and 7–10 min 10% B. Detection of proteins was achieved by Diode Array monitoring at 210 nm. Identification of proteins was achieved by comparing the retention times of sample peaks with isolated standards and quantitation was achieved by comparing the peak areas with those of a standard curve of thaumatin (Sigma, Castle Hill, NSW, Australia). The concentration of phenolic substances of the wines was assessed using absorbance at 280 nm.

### 4.2. Chemicals

Allylamine (AA) (98% Sigma Aldrich, Castle Hill, NSW, Australia), acrylic acid (AcrA) (99% Aldrich), and ethanol (ET) (absolute 99.5% *v*/*v* Ajax Finechem, Thermo Fisher Scientific Australia Pty Ltd., Vic, Australia) were used as received.

### 4.3. Plasma Polymerization 

QCM-D sensors and clean silicon wafers were polymerized in a plasma reactor operated with a 13.56 MHz radiofrequency generator as previously described [37,38]. Allylamine (AA), acrylic acid (AcrA), and ethanol (ET) were used as precursors for plasma deposition in order to generate thin films rich in amine (–NH_2_), carboxyl (–COOH), and hydroxyl (–OH) chemical groups, respectively. Deposition was carried out at a precursors pressure of 0.2 mbar. Allylamine vapor was introduced at 10 sccm, and plasma was applied at 10 W for 4 min. Deposition of acrylic acid was carried out at a flow rate of 0.5 sccm, power of 10 W for 10 min. Whereas, conditions for ethanol deposition were as follows: 4 sccm, 40 W and 4 min. Thickness of the deposited plasma polymers is shown in Figure S10 in Supplementary Materials.

### 4.4. X-ray Photoelectron Spectroscopy (XPS)

X-ray photoelectron spectroscopy was carried on a Kratos Axis Ultra DLD spectrometer (Kratos Analytical Ltd., Wharfside, Manchester, UK) using a monochromatic Al radiation source [19]. The survey scans were performed on each sample at pass energies of 120 eV to identify and quantify the elements present. The C1s and N1s high-resolution spectra were also recorded using pass energy of 20 eV. All binding energies were calibrated with reference to the aliphatic carbon at C1s = 284.8 eV. For processing and curve-fitting, CasaXPS software (ver.2.3.14 Casa Software Ltd., Pasadena, CA, USA) was used.

### 4.5. Water Contact Angle

The contact angle was measured by the sessile drop method using a custom-built contact angle goniometer. A droplet of water was deposited on ppAcrA, ppAA, and ppET surfaces. Images of the droplet were captured using a horizontal digital microscope. Contact angles were determined by drawing the tangent close to the edge of the droplet using a drop shape analysis software ImageJ with the DropSnake plugin (ImageJ 1.46r, Wayne Rasband National Institutes of Health, Washington DC, USA). Five measurements were taken at different locations on the investigated substrates, and an average value was reported. Experiments were performed at room temperature in a clean room (class-100).

### 4.6. Quartz Crystal Microbalance with Dissipation (QCM-D)

The quartz crystal microbalance with dissipation (QCM-D) was used to study wine adsorption. The instrument consisted of an E4 QCM-D unit (Q-Sense, Västra Frölunda, Sweden) coupled with a peristaltic pump (ISM 935, Wertheim, Germany) [19]. During the measurements the QCM liquid chamber was temperature-stabilized to 22 °C and a 0.1 mL/min flow rate for MilliQ water injection was employed until stable frequency and dissipation traces resulted. In the next step, wine was injected into the chamber and remained in contact with the coated gold sensors for 40 min. Rinsing with MilliQ water at the end of adsorption experiments was performed, and the changes in frequency and dissipation were recorded. All experiments with the QCM were repeated four times.

### 4.7. Atomic Force Microscopy (AFM)

Atomic force microscopy analyses were acquired using an NT-MDT SPM (Spectrum Instruments Ltd., Moscow, Russia) with a 100 μm scanner. Images were obtained using non-contact mode in air. Gold coated, silicon nitride non-contact tips with a resonance frequency between 65 and 100 kHz were used. The amplitude of oscillation was 10 nm, and the scan rate was 1 Hz. The AFM parameters were determined using 5 μm × 5 μm images. The AFM software (NTEGRA Prima, NT-MDT SPM, Moscow, Russia) was used to assess the root-mean-squared (RMS) roughness and the peak-to-valley (PTV) distance for the imaged surfaces. The area fraction of wine coverage was determined using ImageJ.

### 4.8. Nanoparticle Tracking Analysis (NTA)

Nanoparticle tracking analysis was performed with a NanoSight NS300 (Malvern Instruments Ltd., Amesbury, Wiltshire, UK), equipped with a sample chamber with a 635 nm laser and a syringe pump. The wine sample was injected in the sample chamber with a sterile 1 mL syringe. A video clip of the wine samples submitted to their natural Brownian motion was captured over 60 s and analysed by the analytical software NanoSight NS300 NTA 2.3 (Malvern Instruments Ltd., Amesbury, Wiltshire, UK). Analysis was performed with a constant and controlled sample flow at room temperature. The samples were measured with manual shutter and gain adjustments. The ‘extended dynamic range mode’ was used as recommended for polydispersed samples. NTA post-acquisition settings were optimized and kept constant between samples, and each video was then analysed to give mean particle size together with an estimate of the concentration.

## 5. Conclusions

Three different precursors, such as allylamine, acrylic acid and ethanol were used in plasma polymerization process to produce thin films rich in amine, carboxyl, and hydroxyl functional groups. The use of QCM-D and AFM allowed quantification of the properties of wine constituents at homogenous surfaces with three different surface chemistries. The results demonstrate that the chemical surface composition generated by plasma polymerization can affect behavior of wine adsorption. The –NH_2_ and –COOH modified surfaces were shown to promote adsorption of constituents from white wine. Among all wine samples, rosé wine exhibited a strong preference for the –OH terminated plasma polymer layer. Red wine adsorbed to the highest extent on acrylic acid surfaces as compared to two other surfaces. These results demonstrate that wine constituents’ adsorption depends on the composition of the wine. It was also found that wine constituents can form poorly hydrated (e.g., on ppAcrA/rosé wine) or highly hydrated layers (e.g., on ppET/white wine) with different characteristics depending on the underlying substrate.

Moreover, the overall results suggest that tuning the surface chemical functionalities is an effective way to modulate wine adsorption. These data may have implications in future design of wine processing technology, such as membranes, and promote the development of tailored sensing systems.

## Figures and Tables

**Table 1:** Adsorbed amount (G), adsorbed volume (V), volume of water in the adsorbed layer (Vw), and proportion of hydration water for white, rosé, and two red wines onto the three plasma polymer surfaces.

Sample	Γ (mg/m^2^) (QCM)	V × 10^−8^ (m^−3^) (QCM) ^1^	V × 10^−8^ (m^−3^) (AFM)	V_w_ × 10^−8^ (m^−3^) Volume of Water (QCM)	% of Water
ppAcrA/White	15.0	1.53	0.54	0.99	65
ppAcrA/Rosé	10.8	1.10	0.50	0.60	55
ppAcrA/Red	9.0	0.92	0.25	0.67	73
ppAA/White	12.2	1.24	0.20	1.04	84
ppAA/Rosé	9.4	0.96	0.18	0.78	81
ppAA/Red	7.0	0.71	0.22	0.49	69
ppET/White	5.4	0.55	0.06	0.49	89
ppET/Rosé	17.5	1.79	0.48	1.31	73
ppET/Red	6.0	0.61	0.16	0.45	74

^1^ Adsorbed volume per unit area data from the QCM-D experiments are calculated using the adsorbed mass per unit area (first column) and the density of wine (980 kg/m^3^).

## Supplementary Materials

Supplementary materials can be accessed at: http://www.mdpi.com/1420-3049/21/10/1394/s1.

## Abbreviations

The following abbreviations are used in this manuscript:

XPS	X-ray photoelectron spectroscopy
QCM-D	quartz crystal microbalance with dissipation monitoring
AFM	atomic force microscopy
NTA	nanoparticle tracking analysis
AA	allylamine
AcrA	acrylic acid
ET	ethanol
pp	plasma polymer
rms roughness	root mean square roughness
PTV	peak-to-valley-distance
ΔPTV	apparent layer thickness
