# Supplementary material for: Adsorption of Wine Constituents on Functionalized Surfaces
Source: Molecules. 2016 Oct 18;21(10):1394. doi: 10.3390/molecules21101394 (PMC6274309; doi:10.3390/molecules21101394)
Supplement: Supplementary file 1 [file molecules-21-01394-s001.pdf]

Supplementary Materials: Adsorption of Wine Constituents on Functionalized Surfaces

Agnieszka Mierczynska-Vasilev and Paul A. Smith

NTA Analysis

Table S1. Particles total concentration, mean size, d90 and standard deviation of wine samples from NTA measurements.

Sample Name	Total Concent Ration (×10⁸/mL)	Mean Size (nm)	SD (nm)	d90 [a]
White	2.04 ± 0.20	190 ± 6.1	89 ± 5.1	327 ± 9.7
Rosé	4.05 ± 0.09	140 ± 2.6	55 ± 3.2	211 ± 6.2
Red	8.41 ± 0.24	292 ± 3.2	70 ± 9.2	454 ± 5.9

[a] 90th percentile under size particle diameter (90% of particles were below this size) SD—standard deviation.

XPS Analysis

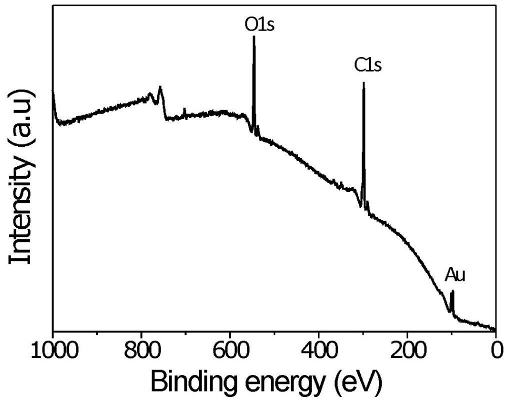

Figure S1. The XPS survey spectra of pp prepared from acrylic acid.

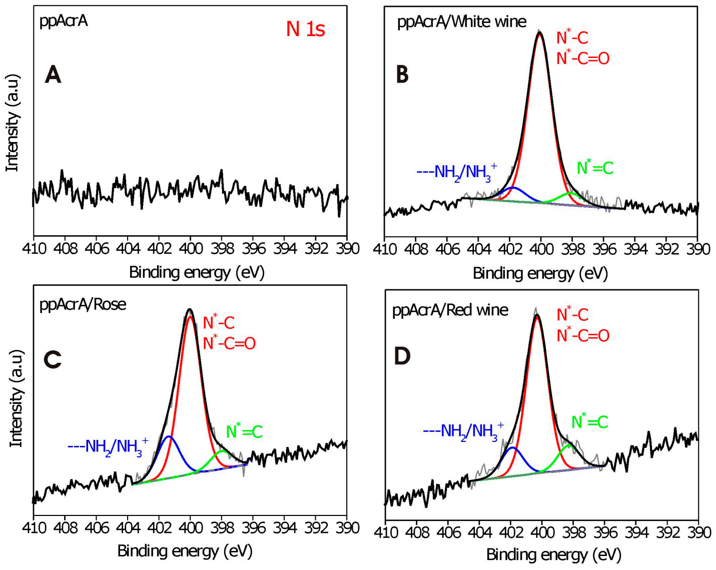

Figure S2. High resolution N1s spectra of different samples: (**A**) Acrylic acid plasma polymerised surface (ppAcrA); (**B**) white wine; (**C**) rosé wine and (**D**) red wine after adsorption on ppAcrA surface. The XPS spectrum of bare ppAcrA has no traces of nitrogen in the N1s spectra, showing that no contamination was present. The ppAcrA coated with different wine exhibited nitrogen peaks.

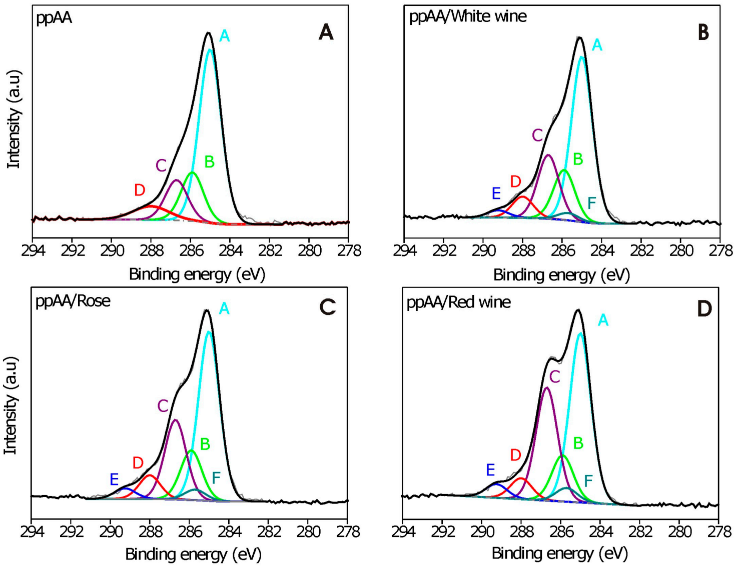

Figure S3. High resolution C1s spectra of different sample: (**A**) allylamine plasma polymerized surface (ppAA); (**B**) white wine; (**C**) rosé wine; and (**D**) red wine after adsorption on ppAA surface.

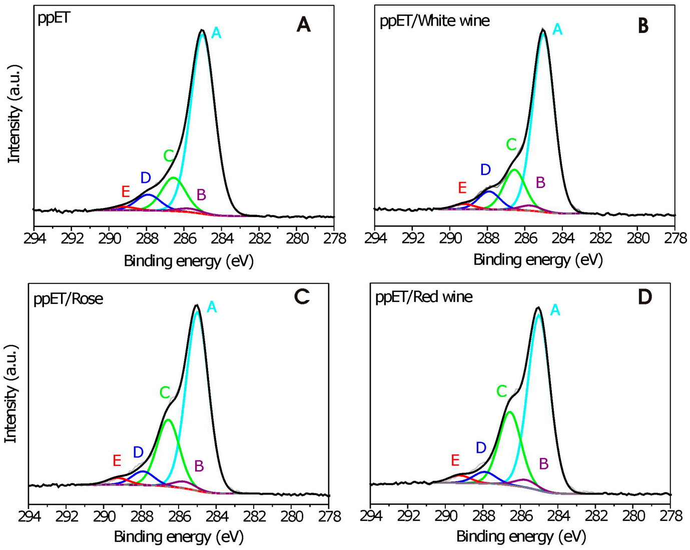

Figure S4. High resolution C1s spectra of different samples: (**A**) ethanol plasma polymerized surface (ppET); (**B**) white wine; (**C**) rosé wine; and (**D**) red wine after adsorption on ppET surface.

QCM-D Measurements

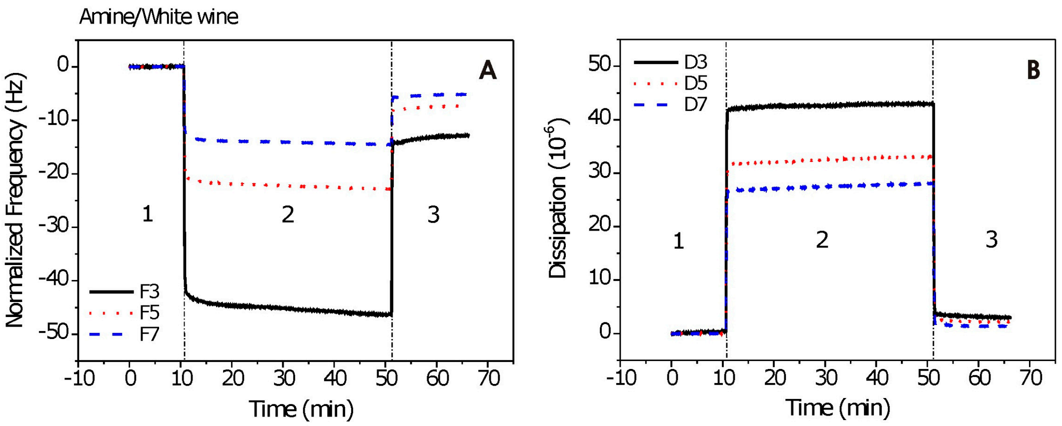

Figure S5. QCM-D data for Riesling white wine binding to allylamine plasma treated QCM sensor. Numbers represent changing flow conditions: (1) baseline in MilliQ-water, (2) exposure to wine, (3) rinsing with water and equilibrating. (**A**) Changes in frequency and (**B**) changes in the simultaneously obtained dissipation values vs. time.

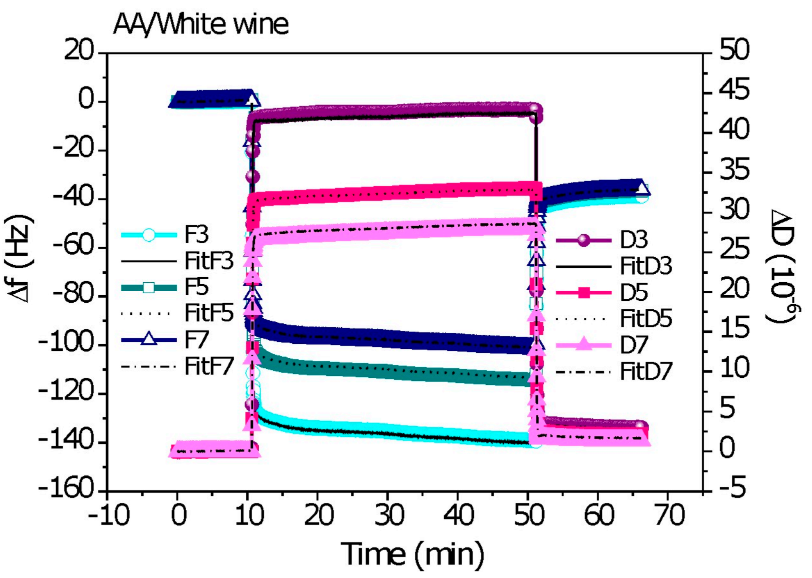

Figure S6. Δf and ΔD vs. time at n = 3 (open and filled circles, respectively), n = 5 (open and filled squares, respectively) and n = 7 (open and filled triangles, respectively) for the QCM data shown in Figure S5. The fit between the Voigt model and the experimental data are shown as filled lines (n = 3), dotted lines (n = 5) and dashed-dotted lines (n = 7).

AFM Measurements

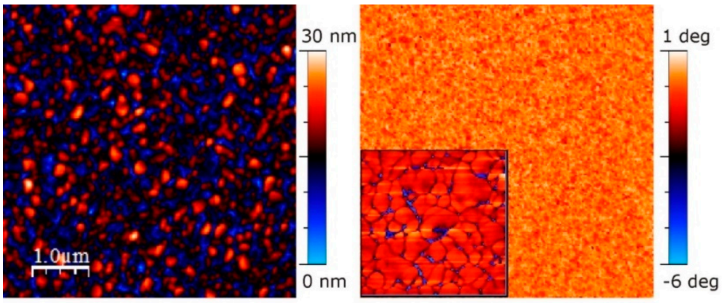

Figure S7. 5 μm × 5 μm AFM height (**left**) and phase (**right**) image of bare QCM sensor. Insert is 2 μm × 2 μm AFM phase image.

Table S2. Rms roughness and peak-to-valley distance of the bare QCM sensor, bare pp surfaces and pp surfaces after wine adsorption.

Sample	rms Roughness (nm)	PTV (nm)	ΔPTV (nm)	Coverage (%)
Bare QCM sensor	3.33	30.31	-	-
Bare AcrA	1.65	15.50	15	100
AcrA/White wine	2.83	31.64	17	32
AcrA/Rosé wine	2.64	31.20	16	31
AcrA/Red wine	1.82	20.97	6	42
Bare AA	1.42	19.11	11	100
AA/White wine	2.70	27.44	8	25
AA/Rosé wine	2.45	25.88	7	26
AA/Red wine	2.29	24.99	6	36
Bare ET	1.47	13.03	17	100
ET/White wine	1.63	19.97	3	20
ET/Rosé wine	2.52	32.27	15	32
ET/Red wine	1.57	19.53	3	53

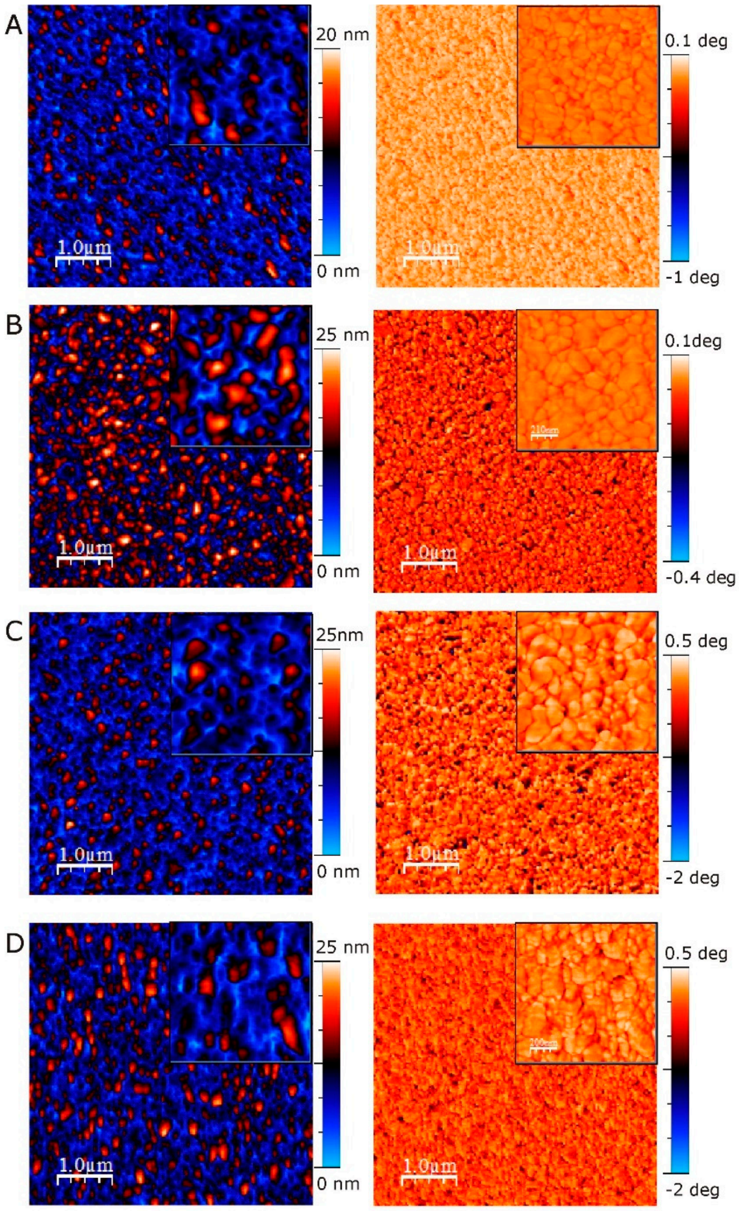

Figure S8. TMAFM images (left: height images; right: phase images) of: (**A**) bare AA pp surface; (**B**) White wine; (**C**) Rosé wine; and (**D**) Red wine on ppAA. Inserts are 2 μm × 2 μm AFM images.

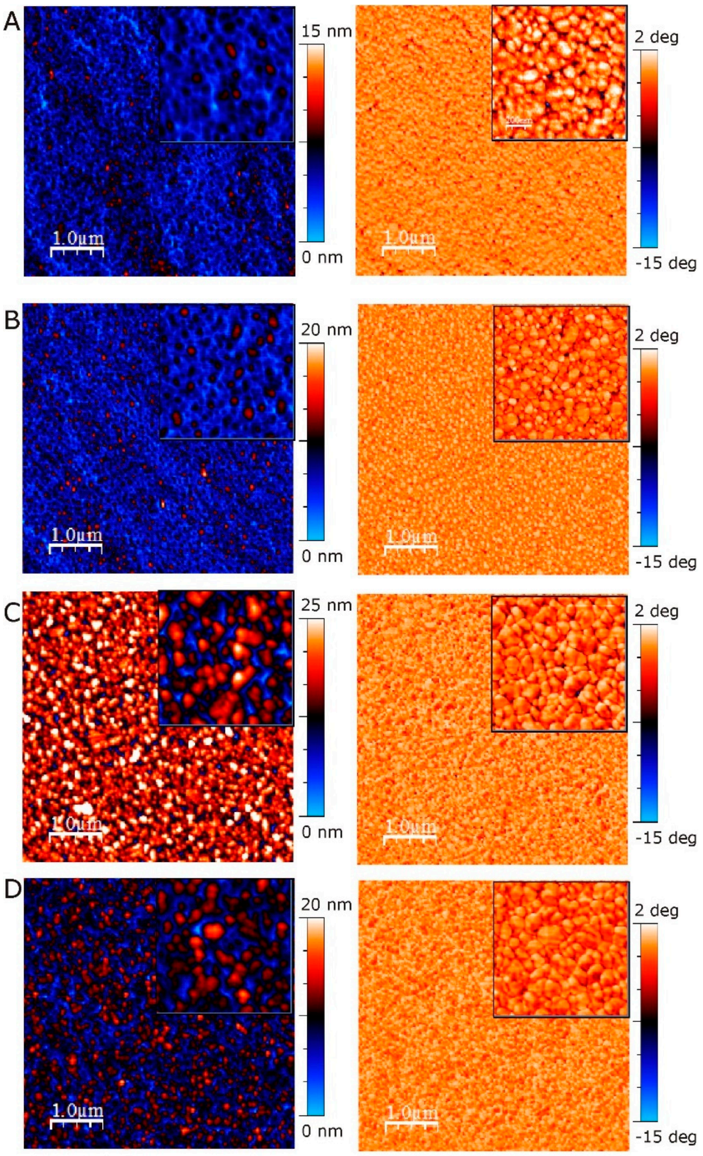

Figure S9. TMAFM images (left: height images; right: phase images) of: (**A**) bare ET pp surface; (**B**) White wine; (**C**) Rosé wine; and (**D**) Red wine on ppET. Inserts are 2 μm × 2 μm AFM images.

Wine Analysis

Table S3. Basic wine chemistry and wine polysaccharides, tannins and proteins content.

Sample	White	Rosé	Red
Volatile acidity as acetic acid	0.3	0.36	0.1
Alcohol (% *v*/*v*)	12.6	11.5	13.4
Specific gravity	0.9911	0.9936	0.9933
pH	3.01	3.59	3.63
Titratable acid pH 8.2 (g/L)	7.4	4.9	6.4
Titratable acid pH 7.0 (g/L)	7.1	4.4	5.9
Glucose + fructose (g/L)	<0.3	0.6	<0.3
Sulfur dioxide (free) (mg/L)	14	35	<5
Sulfur dioxide (total) (mg/L)	70	87	13
Wine tannins (mg/L epicatechin eq.)	nd [a]	56.63	909.05
Phenolic substances (A280)	1.49	2.43	4.32
Wine polysaccharides (mg/L glucose eq.)	222	315	520
Wine proteins (mg/L)	133.72	5.25	nd [b]

[a] Not detected; [b] cannot be measured with the same method, but we know that red wines contain small amounts of free protein.

Thickness Measurements

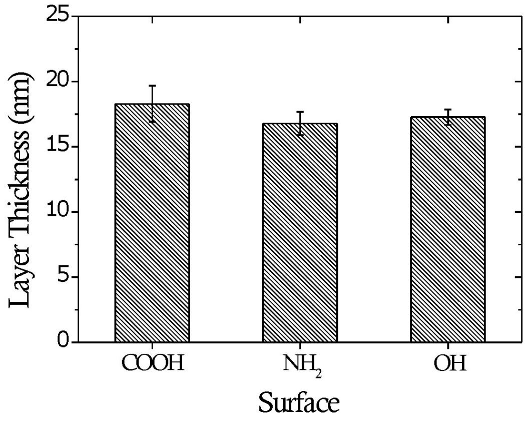

Figure S10. Thickness of the deposited plasma polymers (pps) determined using an ellipsometer (acrylic acid (–COOH), allylamine (–NH_2) and ethanol (–OH)).
